# High salt promotes autoimmunity by TET2-induced DNA demethylation and driving the differentiation of Tfh cells

**DOI:** 10.1038/srep28065

**Published:** 2016-06-21

**Authors:** Haijing Wu, Xin Huang, Hong Qiu, Ming Zhao, Wei Liao, Shuguang Yuan, Yubing Xie, Yong Dai, Christopher Chang, Akihiko Yoshimura, Qianjin Lu

**Affiliations:** 1 Department of Dermatology, Hunan Key Laboratory of Medical Epigenomics, Second Xiangya Hospital, Central South University, Changsha, Hunan, China; 2 Department of Nephrology, Second Xiangya Hospital, Central South University, Changsha, Hunan, China; 3 Changsha Blood Center, Changsha, Hunan, China; 4 Clinical medical research center, the Second Clinical medical college of Jinan University (Shenzhen People’s Hospital), Shenzhen, Guangdong, China; 5 Division of Rheumatology, Allergy and Clinical Immunology, University of California at Davis, Davis, USA; 6 Department of Microbiology and Immunology, Keio University School of Medicine, Tokyo, Japan

## Abstract

Follicular helper T cells (Tfh) have been well documented to play a critical role in autoimmunity, such as systemic lupus erythematosus (SLE), by helping B cells. In this study, high salt (sodium chloride, NaCl), under physiological conditions, was demonstrated to increase the differentiation of Tfh. A high-salt diet markedly increased lupus features in MRL/lpr mice. The mechanism is NaCl-induced DNA demethylation via the recruitment of the hydroxytransferase Ten-Eleven Translocation 2 (TET2). Gene silencing of TET2 obviously diminished NaCl-induced Tfh cell polarization *in vitro*. In addition, the gene expression of sh2d1a, map3k1, spn and stat5b was enhanced after NaCl treatment, consistent with the findings in lupus CD4^+^T cells. However, only spn was directly regulated by TET2, and spn was not the sole target for NaCl. Our findings not only explain the epigenetic mechanisms of high-salt induced autoimmunity but also provide an attractive molecular target for intervention strategies of patients.

Systemic lupus erythematosus (SLE) is a multi-systemic, autoimmune disease that predominately affects women (female to male ratio is 9 to 1) during their reproductive years^1^. SLE is characterized by the presence of a diverse set of autoantibodies in the circulating blood of affected patients^2^ and auto-reactive T and B lymphocytes^3^,^4^. Although the direct cause of SLE remains unidentified, many factors are believed to contribute to immune dysregulation in SLE, including genetic susceptibility, hormones and environmental factors^5^. SLE occurs when an individual with genetic susceptibility to lupus encounters certain variable environmental triggers, which may include diet, toxins, exercise and others.

Increased salt (sodium chloride, NaCl) uptake was recently revealed to promote experimental autoimmune encephalomyelitis (EAE) via the induction of pathogenic Th17 cells^6^. This effect of NaCl occurs via activation of the MAPK/p30 pathway^6^. A prior study by the same group indicated that excess NaCl uptake influences the innate immune system, primarily leading to changes in the activation status of macrophages^7^, and may even affect the suppressive capacity of regulatory T cells^8^. Therefore, one of the aims of the present study is to investigate whether NaCl also affects other immune cells and contributes to the development of SLE.

Many environmental factors have been implicated as triggers of SLE, and it is increasingly apparent that these environmental insults exert their effects via epigenetic mechanisms. External factors which can act synergistically include dietary deficiencies in Vitamin B, folate, methionine (Met), choline and Zn^9^, all of which are necessary to maintain normal DNA methyltransferase 1 (DNMT1) levels^10^. We have previously demonstrated the critical role of DNA hypomethylation in SLE pathogenesis^11^,^12^,^13^,^14^. Therefore, DNA methylation is considered one of the primary mechanisms of environment-triggered immune disorders.

The effect of NaCl on immune cells, including peripheral blood mononuclear cells (PBMCs), CD4^+^T cells, and B cells was evaluated in this study. Surprisingly, among PBMCs, NaCl had no significant effect on Th1, Th2, Th17 and Treg cells but significantly increased the frequency of Tfh cells and promoted Tfh cell differentiation from naïve T cells. Tfh cells are a T helper-cell subset which specializes in in stimulating providing help to B cells in germinal centers (GC). Tfh cells also play a key role in GC formation and the selection of high-affinity B cells in GCs^15^,^16^,^17^. Because SLE is a multi-systemic, autoimmune disease characterized by the presence of a diverse set of autoantibodies in the circulating blood of affected patients^2^ and auto-reactive T and B lymphocytes^3^,^4^, it would be logical that Tfh cells might contribute to the pathogenesis of SLE. Tfh cells are distinguished by surface markers such as CXCR5, PD-1, ICOS and the specific transcription factor BCL-6. CXCR5 guides Tfh cell migration into B-cell follicles, and ICOS delivers activation signals to CD4^+^ T cells when these cells interact with antigen-presenting cells (dendritic cells and B cells) expressing ICOS ligand. ICOS signaling is essential for Tfh cell differentiation^18^. PD-1 is highly expressed on Tfh cells and serves as a negative regulator of Tfh cell differentiation^15^,^16^,^17^,^19^. We have observed a higher percentage of CD4^+^CXCR5^+^PD-1^+^ cells in peripheral blood from SLE patients and CD4^+^CXCR5^hi^PD-1^hi^Bcl-6^+^ frequency was positively correlated with SLEDAI, negatively correlated with ESR and C4 (unpublished data), which is consistent with the results of other studies^20^,^21^,^22^, indicating a critical role of Tfh cells in SLE.

In addition, high-salt diet accelerated the lupus like symptoms on lupus prone (MRL/lpr) mice. However, the effect of a high-salt diet was not as obvious in Balb/c and MRL/mpj mice. Moreover, NaCl-treated CD4^+^T cells displayed DNA hypomethylation and increased DNA hydroxymethylation, potentially correlating with higher levels of the hydroxyltransferases TET2 and TET3. Enrichment of TET2 on spn and stat5b was observed after the addition of NaCl. Silencing TET2 reduced spn gene expression and inhibited Tfh cell differentiation. For the first time our study sheds light on the importance of NaCl in immune disorders and reveals the role of epigenetics in the underlying mechanisms.

## Results

### NaCl increases the frequency of Tfh cells in PBMCs and CD4^+^T cells and promotes Tfh cell differentiation

**Figure 1:**
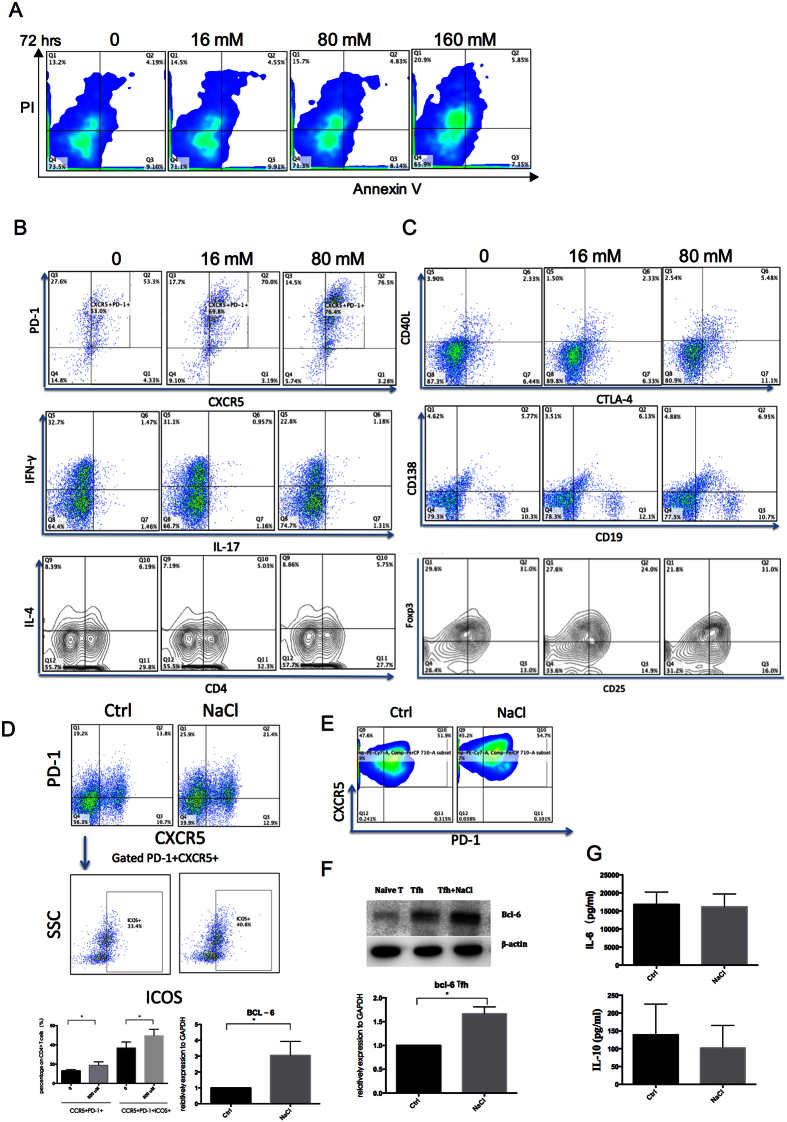
NaCl increases the frequency of Tfh cells among PBMCs CD4^+^T cells and promotes Tfh cell differentiation. Normal PBMCs, CD4^+^T or naïve T cells were cultured with NaCl, and cell surface markers and intracellular molecules were measured by flow cytometry, western blot, qPCR and Bio-plex. (**A**) Apoptosis of PBMCs treated with NaCl (0, 16 mM, 80 mM, and 160 mM) (n = 3). (**B**) Expression of PD-1, CXCR5, IFN- γ, and IL-4 gated on CD4^+^T cells in the presence of anti-CD3 and CD28 stimulation with or without NaCl (n = 3). (**C**) Expression of CD40L, CTLA-4, CD25 and Foxp3 gated on CD4^+^T cells with or without NaCl (n = 3). (**D**) The expression of PD-1, CXCR5, and ICOS on CD4^+^T cells with or without NaCl and mRNA level of BCL-6 (n = 6). (**E**) The expression of ICOS and PD-1 on Day 7 of Tfh cell differentiation and cells were gated on CD4^+^CXCR5^+^ (n = 3). (**F**) Western blot and qPCR data for Tfh differentiation on Day 7 showing the protein and mRNA levels of BCL-6 (n = 3). (**G**) IL-6 and IL-10 production levels from CD4 + T cells cultured without anti-CD3 and CD28 stimulation (n = 3). All flow cytometry figures represent one set of experiments, and each experiment was repeated at least three times on different individuals. Horizontal bars represent the mean ± SEM. *p < 0.05.

To explore the optimal dose for culturing, increasing doses (0, 16 mM, 80 mM, 160 mM) of NaCl were added to normal PBMCs for 72 hr., and apoptosis was evaluated. As shown in Fig. 1A, 16 mM and 80 mM NaCl had no significant effect on cell apoptosis, and 160 mM NaCl did not affect cell viability but we observed a severe cell death at 800 mM (data not shown). In our preliminary study, NaCl concentration less than 16 mM showed no significant effect on CD4^+^T cells, while 80 mM generated stronger stimulation of Tfh cells. In addition, it is reported that 160 mM to 260 mM Na^+^ levels has been found in interstitium and lymphoid tissue^7^,^23^, although normally Na^+^ in plasma is 140 mM. This is the rationale for the choice of 80 millimolar for the bulk of the experiments. Plus with the standard culture medium, which Na^+^ level is approximate 140 mM, the final concentration of NaCl is 220 mM.

To determine which T helper cell subset is affected by NaCl, PBMCs were treated with or without NaCl in the presence or absence of anti-CD3 and CD28 stimulation. Surprisingly, NaCl had no significant effect on IL-17, IFN-γ, and IL-4 production (Fig. 1B) but increased the frequency of Tfh cells (CD4^+^PD-1^+^CXCR5^+^). Moreover, NaCl had little effect on the percentage of Treg (CD4^+^CD25^+^Foxp3^+^) and plasma cells (CD19^+^CD138^+^), and slightly increased the expression of CD40L and CTLA-4 on CD4^+^T cells (Fig. 1C). To further investigate the effect of NaCl on CD4^+^T cells and T cell differentiation, NaCl was added to CD4^+^T cell cultures for 48 hr and naïve T cells during Tfh differentiation. As shown in Fig. 1D, NaCl significantly increased the frequency of the CD4^+^PD-1^+^CXCR5^+^ and CD4^+^PD-1^+^CXCR5^+^ICOS^+^ populations on CD4^+^T cells and the mRNA level of BCL-6, the key transcription factor in Tfh cells^24^. In addition, NaCl increased Tfh cells during Tfh cell differentiation from naïve T cells (Fig. 1E,F). The percentage of CD4^+^PD-1^+^CXCR5^+^ was much higher during Tfh differentiation (Fig. 1E) than in total CD4^+^ T cells (Fig. 1D) due to the Tfh cell polarization condition (IL-6, TGF-β, IL-1β and IL-23). Cytokine production in the supernatant from CD4^+^ T cell cultures was measured by Bio-plex; no effect of NaCl was observed, and most cytokines were under detectable (Fig. 1G). These findings suggest that NaCl increases Tfh cells among PBMCs and CD4^+^T cells and promotes Tfh cell differentiation.

### High salt diet accelerates the progression of lupus in MRL/lpr mice but not MRL/mpj and Balb/c mice

**Figure 2:**
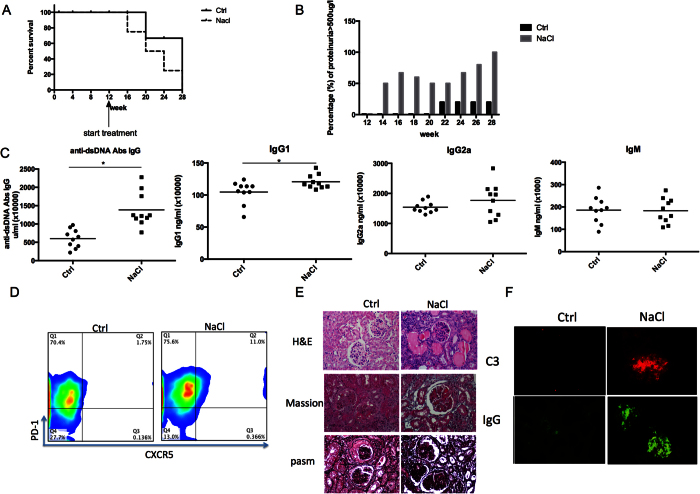
NaCl accelerates the progression of lupus in MRL/lpr mice. Twenty 12-week old MRL/lpr mice were randomly divided into 2 groups that received normal chow and tap water ad libitum (control group) or sodium-rich chow containing 4% NaCl and tap water containing 1% NaCl ad libitum (high-salt group)^6^ until 28 weeks of age. (**A**) Survival of mice. (**B**) Proteinuria. (**C**) Plasma levels of anti-dsDNA antibodies IgG, IgG1, IgG2a and IgM. (**D**) Expression of PD-1 and CXCR5 in CD4^+^ splenocytes in mice treated with or without NaCl. (**E**) Renal histological analysis by H&E, Masson and pasm staining. (**F**) Immunofluorescence histopathological analysis of C3a and IgG deposits in glomeruli. All flow cytometry figures represent one set of experiments, and each experiment was repeated at least 6 times on different mice. The horizontal bars represent the mean ± SEM.

Based on the effects of NaCl on human CD4^+^T cells, we next investigated the effect of a high-salt diet on the mouse *in vivo* system. As shown in Fig. 2, a high-salt diet significantly increased mortality and accelerated the onset of lupus nephritis (Fig. 2A), as evidenced by higher levels of proteinuria (Fig. 2B), plasma levels of anti-dsDNA IgG antibodies and IgG1 (Fig. 2C), lupus-like histological alterations (Fig. 2E), and C3a and IgG deposits in glomeruli (Fig. 2F). A trend of higher frequency of Tfh cells was also observed in splenocytes (Fig. 2D). These results indicate that NaCl can accelerate lupus symptoms in lupus-susceptible mice and suggest that an increase in Tfh cells may be a potential mechanism.

**Figure 3:**
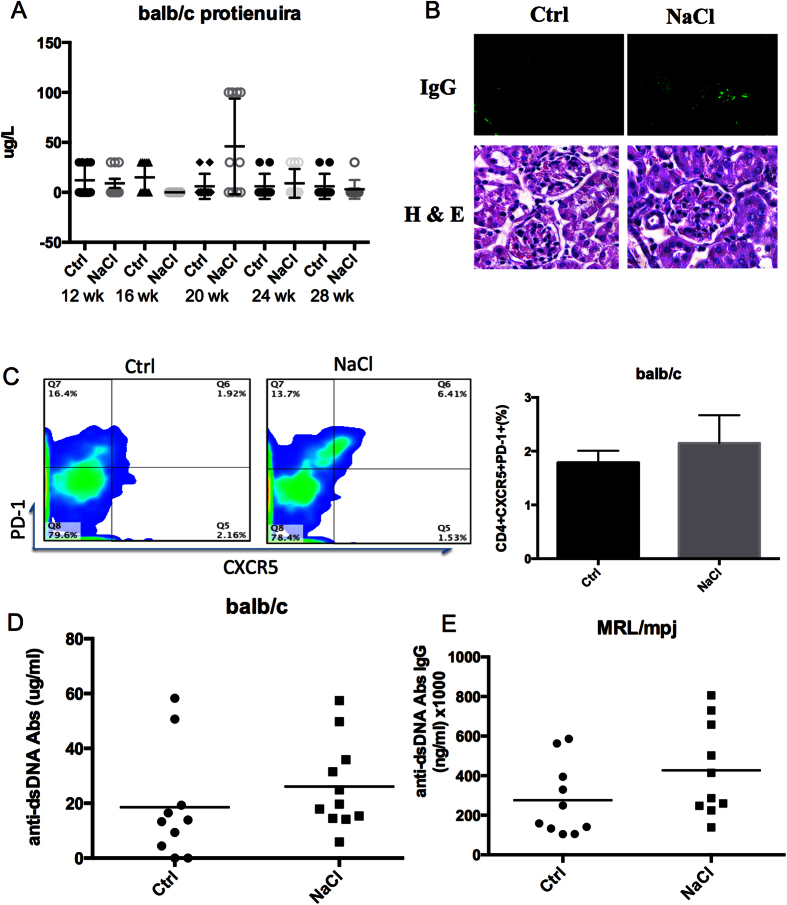
NaCl does not induce or promote lupus-like symptoms in Balb/c and MRL/mpj mice. Twenty 12-week old Balb/c mice were randomly assigned to 2 groups that received normal chow and tap water ad libitum (control group) or sodium-rich chow containing 4% NaCl and tap water containing 1% NaCl ad libitum (high-salt group) until 28 weeks of age. (**A**) Proteinuria levels. (**B**) Immunofluorescence histopathological analysis of IgG deposits and H&E analysis of lupus-like alterations. (**C**) Expression of PD-1 and CXCR5 in CD4^+^ splenocytes. (**D,E**) Level of anti-dsDNA Abs in Balb/c and MRL/mpj mice detected by ELISA. All flow cytometry figures represent one set of experiments, and each experiment was repeated 10 times on different mice. The horizontal bars represent the mean ± SEM.

To further examine the impact of a high-salt diet on normal mice, twenty 12 week-old Balb/c mice were randomly assigned to 2 groups and received normal chow and tap water ad libitum (control group) or sodium-rich chow containing 4% NaCl and tap water containing 1% NaCl ad libitum (high-salt group) until 28 weeks of age. The high-salt diet failed to induce or promote the onset of lupus in Balb/c mice. These mice did not develop proteinuria (Fig. 3A), but did show slightly increased IgG deposits in the glomeruli (Fig. 3B) and enhanced the percentage of Tfh cells in splenocytes (Fig. 3C, *p* > 0.05), and only slight increased anti-dsDNA antibodies (Fig. 3D). Interestingly, the high-salt diet also failed to induce lupus-like symptoms in MRL/mpj mice (n = 20); no obvious increased proteinuria or anti-dsDNA antibodies were observed (Fig. 3E). However loss of body weight and slight renal damage were observed (data was not shown). These findings indicate that a high-salt diet may promote lupus in SLE-susceptible mice but cannot induce SLE in normal mice.

### NaCl induces DNA hypomethylation of CD4^+^T cells and enhances the expression of the hydroxyltransferases TET2 and TET3

**Figure 4:**
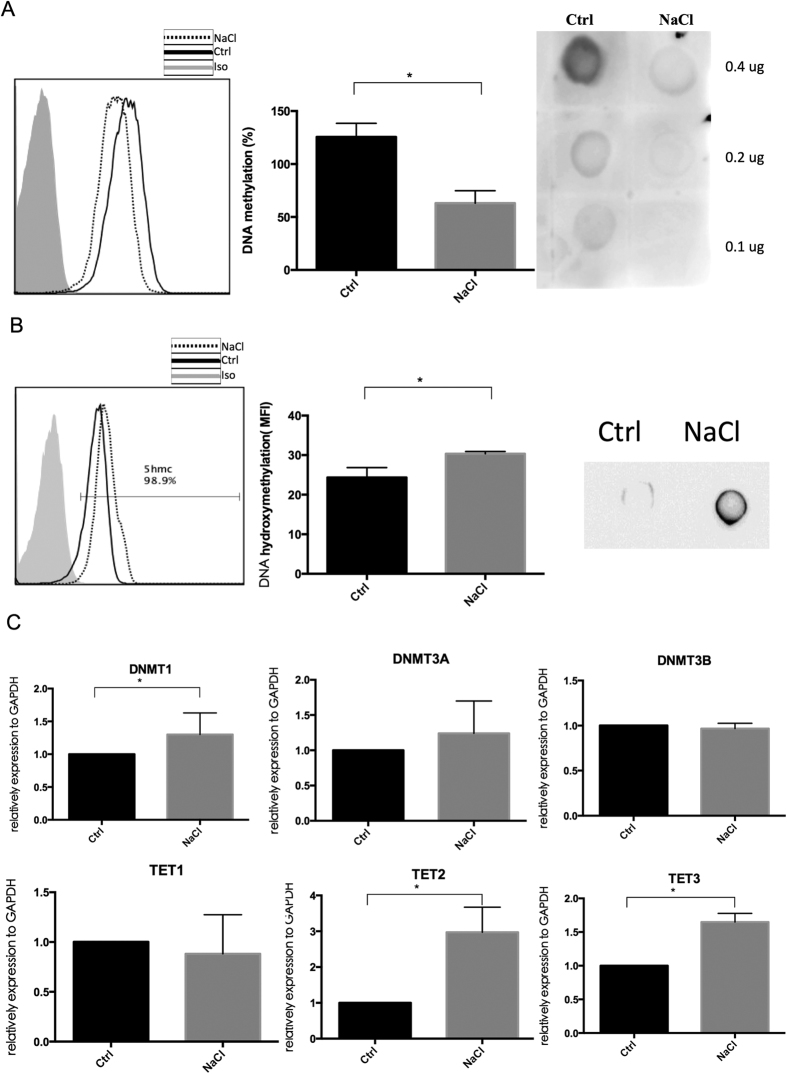
NaCl induces DNA hypomethylation on CD4^+^T cells and enhances the gene expression of TET2 and TET3. Normal human CD4^+^T cells were isolated and cultured with or without NaCl for 48 hr. (**A**) DNA methylation levels were measured by flow cytometry and DNA dot plot (n = 6). (**B**) DNA hydroxymethylation levels were measured by flow cytometry and DNA dot plot (n = 6). (**C**) Gene expression of DNMT1, DNMT3A, DNMT3B, TET1, TET2 and TET3 relative to GAPDH was measured by qPCR and normalized to the control (n = 10). All flow cytometry figures and dot plot figures represent one set of experiments, and each experiment was repeated at least six times on different individuals. The horizontal bars represent the mean ± SEM. *p < 0.05.

To explore the mechanisms of enhancement of Tfh cells in human CD4^+^T cells, we measured DNA methylation and DNA hydroxymethylation levels on normal CD4^+^T cells in the presence or absence of NaCl. As shown in Fig. 4A,B, high-salt-treated CD4^+^T cells exhibited significant DNA hypomethylation and increased hydroxymethylation levels, as confirmed by both flow cytometry and DNA dot plots. These phenomena might be due to an increase in the hydroxyltransferases TET2 and TET3 in the presence of salt (Fig. 4C), specially a dramatic increased level in TET2 (~3 fold). The gene expression of DNMT1 was also increased in high-salt-treated CD4^+^T cells, whereas the differences in DNMT3A and DNMT3B expression levels were not detectable. These data indicate that DNA methylation modification may be involved in the induction of Tfh cells by NaCl.

### TET2 plays an important role in Tfh cell differentiation and NaCl-induced Tfh cell promotion

**Figure 5:**
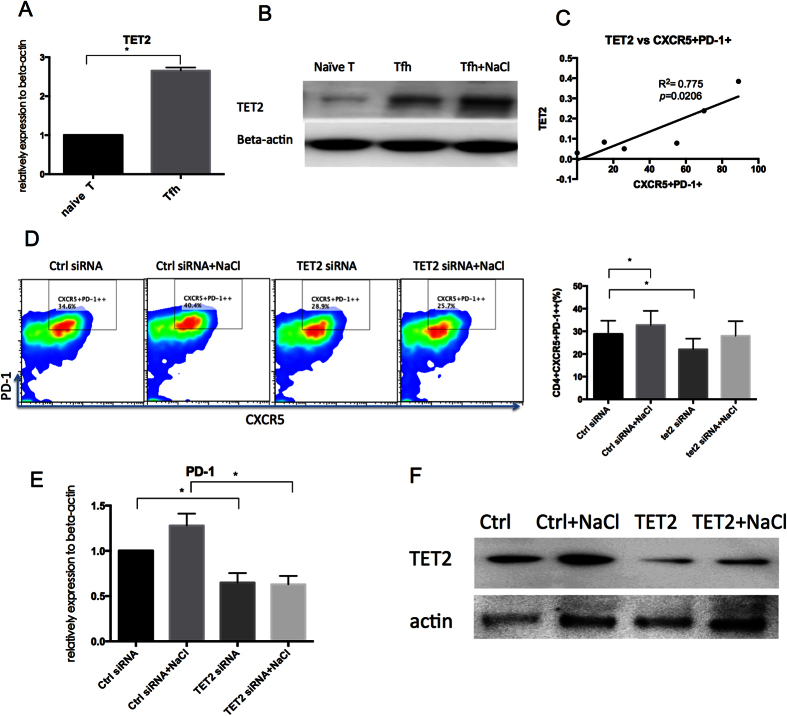
TET2 plays an important role in Tfh cell differentiation and NaCl-induced Tfh cell promotion. (**A**) TET2 gene expression during Tfh cell differentiation (Day 7, n = 6). (**B**) Protein level of TET2 as determined by western blot during Tfh cell differentiation and after NaCl treatment (Day 7, n = 3). (**C**) The correlation of TET2 and CXCR5^+^PD-1^+^ cells during Tfh cell differentiation (Day0, 1, 3, 5, 6, 7, n = 3). (**D**) Normal naïve T cells were transfected with TET2 siRNA or control siRNA and then differentiated into Tfh cells in the presence or absence of NaCl. The percentage of activated Tfh cells (CD4^+^PD-1^++^CXCR5^+^) was detected by flow cytometry (n = 4). Statistical analysis of the Tfh cell percentage as determined by flow cytometry. (**E**) The mRNA level of PD-1 (n = 3). (**F**) The protein level of TET2 in Tfh cells after transfection (n = 3). All flow cytometry figures and western blot figures represent one set of experiments, and each experiment was repeated at least three times on different individuals. The horizontal bars represent the mean ± SEM. *p < 0.05.

To determine if TET2 participates in Tfh cell differentiation, the mRNA and protein levels of TET2 were determined. As shown in Fig. 5C, compared with normal naïve T cells, Tfh cells on Day 7 exhibited a significant increase in TET2 protein levels that was further enhanced by the addition of NaCl. Moreover, the mRNA level of TET2 was positively correlated with the percentage of CXCR5^+^PD-1^+^ cells during Tfh cell differentiation, suggesting that TET2 plays a role in Tfh cell differentiation. The role of TET2 was confirmed by using TET2 siRNA to knock down TET2 expression during Tfh cell differentiation. TET2 siRNA dramatically decreased the percentage of Tfh cells (CXCR5^+^PD-1^++^) on Day 7 (Fig. 5D). The addition of NaCl slightly increased the percentage of CXCR5^+^PD-1^++^ cells (*p* = 0.07), but had no effect on PD-1 mRNA level (Fig. 5E), indicating that TET2 played an important role in Tfh cell differentiation and that NaCl promoted Tfh cell differentiation in a TET2-dependent manner. The increased number of CXCR5^+^PD-1^++^ cells induced by NaCl may be a result of slightly increased TET2 protein levels (Fig. 5F) after siRNA transfection.

### NaCl enhances the expression of map3k1 and spn and induces DNA hypomethylation of genes that are involved in the immune response pathway

**Figure 6:**
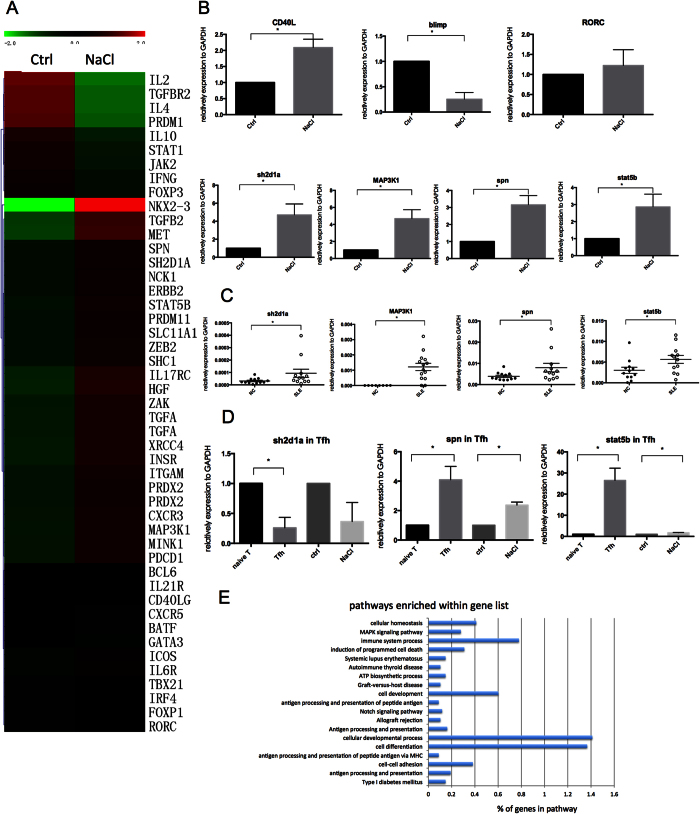
NaCl enhances the expression of map3k1 and spn and induces DNA hypomethylation of genes that are involved in the immune response pathway. (**A**) Heat map: These genes were selected based on their functions in T helper cell subsets (more than 2-fold change, *p* < 0.05). (**B**) Gene expression on normal CD4^+^T cells (with and without NaCl treatment for 48 hr, n = 10). (**C**) Gene expression on CD4^+^T cells from normal controls (n = 12) and SLE patients (n = 12). (**D**) Gene expression during normal Tfh cell differentiation (n = 3). (**E**) Methylation map: GO analysis beta dif <−0.02, p < 0.05. All flow cytometry figures and dot plot figures represent one set of experiments, and each experiment was repeated at least three times for different individuals. The horizontal bars represent the mean ± SEM. *p < 0.05.

To further explore the mechanism of NaCl-induced Tfh cell promotion, a gene expression map and DNA methylation map were constructed for control and NaCl-treated human CD4^+^T cells. Compared with control cells, the high-salt-treated cells exhibited decreased gene expression of Blimp, IL-4, IL-10, IFN-γ, and IL-2 (2-fold change, *p* < 0.05) and enhancement of T helper cell subset differentiation genes, such as ICOS, PDCD1, STAT1, and Sh2d1a, and other genes involved in T cell differentiation and activation (2-fold change, *p* < 0.05, Fig. 6A). However, transcription factors that are essential for T helper cell differentiation, such as BCL-6, TBX21, GATA3, FOXP3 and RORγt, did not exhibit significant changes. However, BCL-6 showed significant changes in 10 normal CD4^+^ T cells by the high-salt treatment, which was detected by QPCR (Fig. 6D).

Among the genes involved in T cell differentiation and activation that were increased by NaCl by more than 2-fold, sh2d1a, map3k1, spn and stat5b attracted our attention and were validated in 10 normal CD4^+^T cells (Fig. 6B). In addition, NaCl treatment increased the expression of CD40L at the protein (Fig. 1C) and mRNA levels in CD4^+^ T cells, but these increases did not differ significantly from the gene expression map (Fig. 6B). Furthermore, blimp, which promotes Th17, Th1 and Th2 and represses Tfh cell differentiation^25^, was also validated in 10 NaCl-treated CD4^+^T cells.

Accordingly, the mRNA levels of sh2d1a, map3k1, spn and stat5b were measured in NC vs SLE CD4^+^T cells and naïve vs Tfh cells (with or without NaCl). As shown in Fig. 6C, the gene expression of sh2d1a, map3k1, spn and stat5b was significantly increased in SLE patients compared with NC subjects. However, no correlation was observed between the expression of these genes and the SLEDAI (data not shown). During Tfh differentiation, the expression of spn and stat5b was enhanced, and the addition of NaCl further increased these levels (Fig. 6D), indicating that these genes might be involved in Tfh differentiation and respond to NaCl treatment. The data from the methylation map revealed that NaCl induced DNA hypomethylation of genes (Fig. 6E, GO analysis beta dif <−0.02, p < 0.05) mainly involving cell differentiation, cellular development process, and immune system process. These data suggest that NaCl may activate cell differentiation and the immune system by inducing DNA hypomethylation.

### NaCl treatment enriches TET2 on spn and stat5b, and TET2 silencing inhibits spn expression

**Figure 7:**
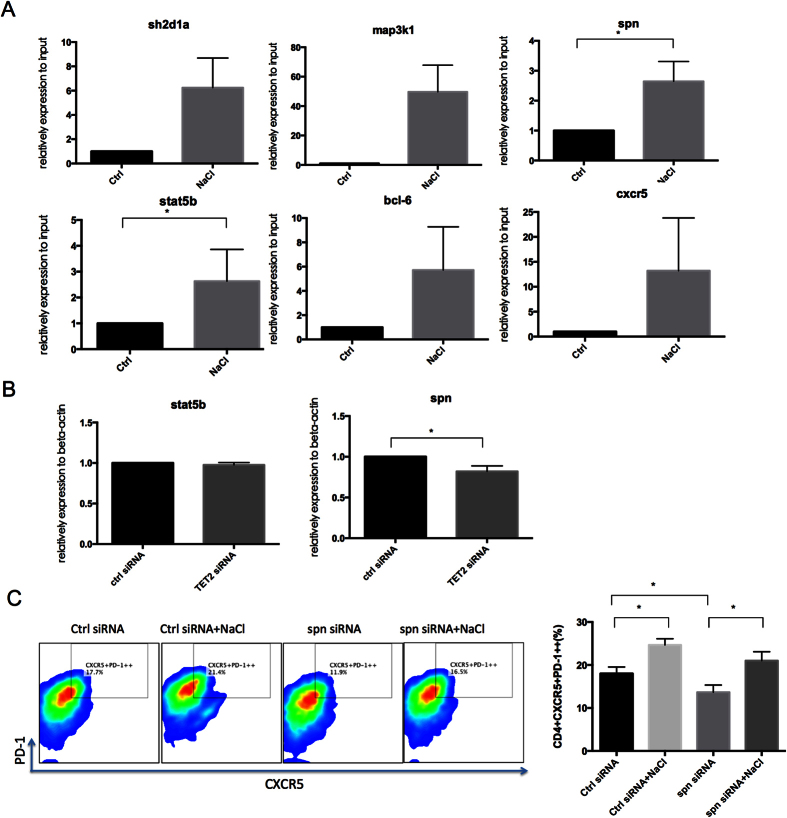
Enrichment of TET2 on spn and stat5b after NaCl treatment and TET2 regulation of spn expression. (**A**) Normal human CD4^+^T cells were isolated and cultured with or without NaCl for 48 hr. Then, the cell lysates were immunoprecipitated with 2 ml of anti-TET2 antibody. DNA was eluted, and qPCR of sh2d1a, map3k1, spn, CXCR5, BCL-6 and stat5b was performed (n = 6). (**B**) TET2 siRNA was applied to knock down TET2 during Tfh differentiation in the presence or absence of NaCl. The mRNA levels of spn and stat5b were evaluated on Day 5 (n = 3). (**C**) Normal human CD4^+^T cells were isolated, transfected with spn siRNA and cultured with or without NaCl under Tfh cell differentiation conditions. The percentage of CD4^+^CXCR5^+^PD-1^++^ Tfh cells was detected by flow cytometry (n = 3). The horizontal bars represent the mean ± SEM. *p < 0.05.

To further explore the interaction between TET2 and NaCl-increased genes (sh2d1a, map3k1, spn, stat5b, BCL-6), ChIP-qPCR was performed. As shown in Fig. 7A, TET2 enrichment was observed on spn and stat5b (n = 6, *p* < 0.05), indicating that increased expression of spn and stat5b might be associated with the enrichment of TET2 after NaCl addition. Then, the expression of these genes was evaluated after TET2 silencing. Moreover, TET2 silencing only affected spn expression statistically (Fig. 7B). To further silence spn during Tfh differentiation, spn siRNA reduced the CXCR5^+^PD-1^++^ cells, however, the addition of NaCl dampened its inhibiting effects (Fig. 7C), suggesting that spn regulated Tfh cell differentiation and served as one but not the only target for NaCl.

## Discussion

Accumulating evidence supports a role of high salt in immune disorders, including EAE^6^,^26^ and xenogeneic graft-versus-host disease (GVHD) model^8^. Pathogenic Th17 cells, which are important for development of EAE, are promoted by activation of the p38/MAPK pathway by NaCl^6^. High salt attenuates the function of regulatory T cells (Tregs) and induces IFN-γ-producing Tregs mediated by serum/glucocorticoid-regulated kinase (SGK1)^8^, thus aggravating GVHD. Specific activation is observed on macrophages induced by NaCl-triggered activation of p38/cFos and/or Erk1/2/cFos pathways^27^, as well as NF-kB and MAPK signaling pathways^26^, promoting lung inflammation and central nervous system autoimmunity, respectively. These findings indicate that high salt promotes autoimmunity via pro-inflammatory responses.

In this study, we observed an enhanced frequency of Tfh cells rather than Th17^6^ and Treg cells^8^. These might due to the different cell population (PBMCs and CD4^+^T cells) we investigated. In Th17 cell investigation, purified CD4^+^ naïve T cells were the target cells. Unlike the mixture of PBMCs and CD4^+^T cells, the differentiation of Th cells would not be affected by other cells, such as antigen presenting cells and memory T cells. However, in physiological condition, cells in lymphoid organs are more like a mixture of cells rather than purified naïve T cells. As shown in Fig. 1, NaCl significantly increased the percentage of CD4^+^CXCR5^+^PD-1^+^ cells and the size of the ICOS^+^ population, indicating that NaCl enhanced the number and function of Tfh cells. The role of Tfh cells in SLE has been well documented. In the Roquin^san/san^ mouse model, the pathogenic roles of Tfh have been clearly demonstrated to involve impaired function of the post transcriptional repressor Roquin, which causes the development of a lupus-like syndrome. This process is mediated by excessive Tfh cell and GC responses^28^,^29^,^30^, and Tfh cells also contribute to lupus in MRL/lpr mice^31^,^32^. Additionally, multiple lines of evidence indicate a pathogenic role of Tfh cells in human lupus, such as alterations in the phenotype of circulating Tfh (cTfh) cells^32323123^ and increases in their numbers^20^,^21^,^22^. The differential expression of ICOS and PD-1 on human cTfh cell subsets further defines distinct subpopulations: an activated subset (ICOS^+^PD-1^++^) and two quiescent subsets (ICOS^-^PD-1^+^ and ICOS^-^PD-1^−^)^33^. In SLE, the activated subset (ICOS^+^ or PD-1^++^) of cTfh cells is increased and is positively correlated with serum autoantibody titers and disease activity and/or severity^25^. We previously observed a significant increase in the PD-1^++^CXCR5^+^ population in SLE (unpublished data) as well as an increase in PD-1^++^ Tfh cells after NaCl treatment (Fig. 1), indicating activation of the Tfh subset in SLE and high-salt conditions. In addition, MRL/lpr mice that received a high-salt diet exhibited accelerated disease progression, with a higher proportion of Tfh cells in the spleen (Fig. 2). However, Balb/c and MRL/mpj mice failed to develop severe lupus-like symptom, even though IgG deposits and proteinuria were slightly elevated in the high-salt group (Fig. 3), suggesting that the risk of lupus is influenced by complex genetic and environmental contributions.

Epigenetic modulation is considered one of the mechanisms involved in NaCl promotion of Tfh cell differentiation, based on previous reports that Vitamin B, folate, methionine (Met), choline and Zn^9^ are required to maintain a normal level of DNMT1^10^. We observed enhancement of DNMT1 in the high-salt group. However, global DNA methylation levels were dramatically attenuated by NaCl (Fig. 4), and the significant increase in TET2 levels might be a contributor. In addition, the high-throughput MDIP-seq data suggest that NaCl treatment mainly reduces DNA methylation levels of genes involved in T cell activation and differentiation (Fig. 5). TET2 proteins catalyze the conversion of 5-methylcytosine (5 mC) to 5-hydroxymethylcytosine (5 hmC) to mediate DNA demethylation. Accumulating evidence indicates that TET2 controls the methylation and differentiation of Th1 and Th17 cells by recruitment on lineage-specific cytokine genes, such as *Il6* and *Infg*^34^. Although TET2 is a negative regulator in Tfh cells in mice^35^, our *in vitro* results for human Tfh cells strongly support a positive role of TET2 in Tfh cell differentiation by promoting PD-1 and BCL-6 expression (Fig. 6). A direct interaction of TET2 and BCL-6 was also observed by ChIP-seq (Fig. 7), suggesting a regulatory role of TET2 in Tfh cell differentiation. However, no difference in IL-21 expression and no TET2 enrichment on the *Il21* gene were observed.

The data from high-throughput sequencing suggests that several genes involved in T cell differentiation and activation are activated by high-salt treatment, such as sh2d1a, map3k1, spn and stat5b. The expression of these four genes was also higher in lupus CD4^+^T cells (Fig. 6). TET2 silencing revealed that only spn interacts with TET2 and is affected by TET2 silencing. Spn (CD43) is a transmembrane glycoprotein with an elongated extracellular domain and a highly conserved intracytoplasmic tail^36^. Numerous ligands of spn have been identified, such as ICAM-1, MHC-I, E-selectin, and galectin-1, and complex glycosylation patterns of spn have been observed in pathogens such as viruses, *M. tuberculosis* and *T. cruzi*, indicating that spn can act as a pathogen recognition receptor. The colligation of spn with the T cell receptor promotes T cell proliferation and IL-2 production by enhancing ERK activation^37^. We observed that spn played an essential role in T cell differentiation. Naïve T cells in which spn was knocked down by siRNA exhibited no response to CD3CD28 activation. Thus, silencing of spn was conducted in CD4^+^T cells instead. After silencing, the Tfh cell population decreased, and NaCl reversed these effects (Fig. 7), suggesting that spn played a critical role in Tfh cell differentiation and NaCl might promote Tfh cells and not exclusively through spn.

In the present study, we identified a high-salt diet as an environmental factor that promotes SLE by inducing Tfh cell differentiation. However, in the mouse model, the effects of a high-salt diet were much more complicated than that in the *in vitro* system. This difference may be attributable to Tfh cells. Pathogenic Th17 cells, activated macrophages, and function-reduced Treg cells might work together to contribute to SLE progression, but Tfh cells play a significant role in modulating the epigenetic effects of the environment. *In vivo* effects of high-salt in lupus mice were not unlikely attributed to increased GFR due to a saline diuresis because the high-salt diet failed to have similar results in normal mice. Although the higher concentration of NaCl was observed in lymphoid tissues in mice, the hypertonicity might also contribute to the immune cells. But at least, our data indicate that lupus patients would be better to reduce salt in-take and the underlying mechanism might provide new therapeutic target for SLE, with interference TET2 and spn to attenuate pathogenic Tfh cells.

## Materials and Methods

### Subjects and mice

A total of 12 SLE patients who fulfilled at least 4 of the SLE classification criteria of the American College of Rheumatology were recruited from outpatient clinics in the Second Xiangya Hospital of Central South University^38^. Lupus disease activity was assessed using the SLE Disease Activity Index (SLEDAI)^39^. The characteristics of SLE patients are shown in S Table 1. A total of 54 healthy controls were recruited from the medical staff at the Second Xiangya Hospital and Changsha Blood Center. The information of healthy control is shown in S Table 1 and 2. Patients and controls were matched for age and sex in all experiments. The human sample study was approved by Ethics Committee of Second Xiangya Hospital, Central South University, and the methods were carried out in accordance with the approved guidelines. Written informed consent was obtained from all subjects.

MRL/lpr, MRL/mpj and Balb/c mice were purchased from Jackson Laboratory (USA). Twenty 12-week-old female MRL/lpr, MRL/mpj and Balb/c mice were randomized into 2 groups, either receiving normal chow and tap water ad libitum (control group) or sodium-rich chow containing 4% NaCl and tap water containing 1% NaCl ad libitum (high-salt group) every day. Mouse studies were approved by the Animal Care and Use Committee of Second Xiangya Hospital, Central South University, and the methods were carried out in accordance with the approved guidelines.

### Cell isolation, culture and differentiation

PBMCs were separated from the peripheral blood of healthy controls and SLE patients by density gradient centrifugation (GE Healthcare, Switzerland). CD4^+^T and B cells were isolated by positive selection using Miltenyi beads according to the manufacturer’s instructions (Miltenyi, Germany). CD4^+^CDRA^+^/RO^−^ naïve T cells were isolated by negative selection by Miltenyi beads according to the manufacturer’s instructions. The purity of the enriched subset was validated by flow cytometry and was generally higher than 95%.

NaCl (Sigma, USA) (16 mM, 80 mM and 160 mM) was added to PBMCs and CD4^+^T cells (5 × 10^5^ cell ⁄ml) for 48 hr. In some experiments, NaCl was added to PBMCs in the presence of anti-CD3 and anti-CD28 (1 μg/ml, eBioscience, USA), and Golgi stop (BD Pharmingen, USA), PMA (50 ng/ml) and ionomycin (750 ng/ml, Sigma, USA) were added for last 4 hr of culture. Cells and supernatants were harvested for subsequent analysis.Tfh cell differentiation: CD4^+^CDRA^+^/RO^−^ naïve T cells were seeded in anti-CD3 antibody (1 μg/ml) pre-coated 24-well plates (2.5 × 10^5^ cell ⁄ml) with or without NaCl in the presence of anti-CD28 antibody (1 μg/ml), IL-6 (5 μg/ml), TGF-β (1 μg/ml), IL-1β (10 μg/ml) and IL-23 (10 μg/ml) for 7 days. The medium was refreshed on Day 3 and Day 5^40^. The cells were harvested for subsequent analysis.

### Flow cytometry

To examine the expression of surface markers and intracellular molecules, cells were incubated with FcR blocking reagent (Miltenyi, Germany) for 10 min followed by primary antibodies on ice in the dark for 30 min. The antibodies used for surface marker analysis included anti-human CD4-FITC, CXCR5-PE, PD-1-PE-cy7, BCL-6-APC, CD25-PE, CD19-FITC, CD138-PE (BD Pharmingen, USA), and anti-mouse CD4-FITC, CXCR5-APC, PD-1-PE, and CD138-PE (eBioscience, USA). For intracellular staining, cells were cytofixed and cytopermed using the CytofixCytoperm Plus kit (BD Pharmingen, USA) and stained with intracellular antibodies, including anti-human IL-17-APC, IFN-γ-PE, IL-4-PE-cy5, Foxp3-APC (BD Pharmingen, USA), 5-mC and 5-hmC (Abcam, USA) for an additional 30 min on ice in the dark. For some experiments, apoptosis was detected using a FITC Annexin V Apoptosis Detection Kit II (BD Pharmingen, USA). Data were acquired by flow cytometry (BD, Canto II, USA) and analyzed using FlowJo (Tree Star, USA).

### Cytokine detection by Bio-plex

Supernatants were harvested from cultures and stored at −80 °C for subsequent cytokine measurement. Cytokines including IL-4, IL-6, IL-17A, IL-17F, IL-21, IL-23, IFN-γ and IL-10 were measured by a Bio-Plex Pro human Th17 cytokine assay, and data were recorded on a Bio-plex 200 system (Bio Rad, USA).

### DNA dot plot

DNA was extracted from CD4^+^ T cells by a QIAamp DNA Mini kit (QIAGEN, USA). The dot plot procedure was performed as described by Abcam. Briefly, a pencil was used to draw a grid on the nitrocellulose membrane (Bio-Rad, USA). A narrow-mouth pipette tip was used to spot 2 μl of DNA samples onto the nitrocellulose membrane at the center of the grid. The area the solution penetrated was minimized, and the membrane was allowed to dry. Non-specific sites were blocked by soaking in 5% BSA (Sigma, USA) in TBS-T for 1 hr at room temperature. The membrane was then incubated with 5-mC and 5 hmC primary antibody (Abcam, USA, 1:2000) dissolved in BSA/TBS-T for 30 min at room temperature. The membrane was washed three times with TBS-T and incubated with a HRP-conjugated secondary antibody (Abcam, USA, 1:4000) for 30 min at room temperature. The membrane was washed three times with TBS-T, incubated with ECL reagent for 1 min, covered with plastic wrap, and exposed to X-ray film in the dark.

### Quantitative PCR

Total RNA was extracted from cells using Trizol reagent (Invitrogen, USA). The sample was reverse-transcribed with the PrimeScript®RT reagent kit with gDNA Eraser (TaKaRa Biotech Co., China) using 1 μg of total RNA according to the manufacturer’s instructions. The reaction mixture contained 2 μl of cDNA, 10 μl of SYBR Premix Ex Taq^TM^ (TaKaRa Biotech Co., China), and 400 nM sense and antisense primers to a final volume of 20 μl. Transcripts were measured using a Rotor-Gene3000 (Corbett Research, NSW, Australia) thermocycler. The quantity of gene expression was calculated using the 2^−ΔCt^ method and normalized to GAPDH. Primers for BCL-6, DNMT1, TET2, TET3, Blimp, CD40L, STAT5B, SPN, SH2D1A, and MAP3K1 were purchased from Life Technologies, USA.

### Western blotting

Proteins in cell lysates were quantified by the Bradford assay (HyClone-Pierce, USA) followed by 12% vertical dodecyl sulfate-polyacrylamide gel electrophoresis and transferred to nitrocellulose membranes (Millipore, USA). The membrane was blocked in TBS/5% skim milk and then incubated with mouse anti-human BCL-6 or TET2 monoclonal antibody (Santa Cruz, USA) for 1 h, followed by HRP-rabbit anti-mouse IgG antibody (Santa Cruz, USA). Proteins were detected with an ECL Western blot detection kit (Thermo Scientific, USA). Quantification of BCL-6 or TET2 was normalized to β-actin by densitometry.

### Chromatin immunoprecipitation (ChIP)-qPCR

To explore the possible molecular mechanisms of NaCl in regulating immune cells, we analyzed the binding of TET2 at *sh2d1a, stat5b, map3k1* and *spn*. Chromatin immunoprecipitation (ChIP) assays were performed with a ChIP kit (Millipore, USA). According to the protocol, CD4^+^ T cells, with or without NaCl treatment, were fixed in 1% formaldehyde and then lysed for 10 min on ice. Chromatin was sheared by sonication. After preclearing with protein-G agarose beads, the cell lysates were immunoprecipitated overnight at 4 °C with 2 ml of anti-TET2 antibody (Millipore, USA). Then, protein G agarose beads were added and the resulting mixture was rotated for 1 hr at 4 °C. After washing and elution, cross-links were reversed by heating at 65 °C for 4 h. The eluted DNA was purified, and the samples were analyzed by qPCR with input DNA (total chromatin) as an endogenous control. SYBR Premix Ex Taq^TM^ was used to detect the enrichment of *sh2d1a, stat5b, map3k1* and *spn* (primers were purchased from life technology, USA).

### siRNA silencing

Transfection was performed using Human T Cell Nucleofector kits and nucleofector (Amaxa, Germany). Control-siRNA, TET2-siRNA, and spn-siRNA were purchased from Life technology, USA. T cells transfected with control-siRNA or siRNA were treated with or without 80 mM NaCl for 48 hr. The cells and supernatants were harvested for subsequent analysis.

### High throughput sequencing and MDIP-seq

NaCl (Sigma, USA) was added to CD4^+^T cells (5 × 10^5^ cell ⁄ml) for 48 hr. DNA and RNA was extracted and high throughput sequencing and MDIP-seq were conducted by Shanghai Biotechnology Corporation, China.

### Animal experiments

#### Proteinuria

Proteinuria was semi-quantitatively assessed biweekly using Albustix assay strips (Siemens Healthcare Diagnostics). The levels of urinary albumin were scored as follows: 0 = trace; 1 = 30–99 mg/d; 2 = 100–299 mg/dl; 3 = 300–1999 mg/dl; and 4 =>2000 mg/dl.

Anti-dsDNA antibody detection by ELISA: Mouse blood samples were collected from the lateral saphenous vein biweekly. A 100-μl volume of blood was collected in pre-calibrated micro-hematocrit tubes with heparin and then centrifuged at 13000 rpm for 10 minutes. Plasma was collected, aliquoted and stored for further use. Anti-dsDNA antibody levels were detected by mouse anti-dsDNA IgG, IgG1, IgG2a, and IgM ELISA Kit (Alpha Diagnostic Intl, USA). The OD value was recorded by enzyme-labeled instrument, Enspire, USA.

#### Histopathology

Mice were sacrificed at 28 –week-age, and kidneys were collected, fixed in 10% buffered formalin, and embedded in paraffin. Hematoxylin and eosin (H&E), Masson and pasm staining were performed. Glomerular injury was observed under a microscope (Leica, Germany).

#### Immunofluorescence histopathology

Kidneys were snap-frozen in liquid nitrogen and embedded in Optimal Cutting Temperature compound (Leica, Germany). After blocking with 1% BSA, frozen sections (3 μm thick) were stained with Alexa 488-conjugated anti-mouse IgG (1:100, Biolegend, USA) or rabbit anti-mouse C3 antibody (1:100, BD Pharmingen, USA) in a dark, humidified chamber for 1 hr and washed with PBS. For C3 detection, sections were incubated with a DyLight 594-conjugated goat anti-rabbit secondary antibody (1:500, Genecopoeia, USA) for 1 hr, washed with PBS, mounted and visualized by fluorescence microscopy.

### Statistical analysis

All diagrams and graphs report cumulative data as means ± standard error of the mean (SEM). Distributions of means were analyzed with non-parametric tests (SPSS 16.0, USA). Differences within individual treatments were analyzed by paired t-test. A *p*-value < 0.05 was considered statistically significant.

### Study approval

Patients and controls were matched for age and sex in all experiments. Written informed consent was obtained from all subjects. Mouse studies were approved by the Animal Care and Use Committee of Second Xiangya Hospital, Central South University.

## Additional Information

**How to cite this article**: Wu, H. *et al*. High salt promotes autoimmunity by TET2-induced DNA demethylation and driving the differentiation of Tfh cells. *Sci. Rep.* **6**, 28065; doi: 10.1038/srep28065 (2016).
